# Stereotactic Ablative Radiation Therapy (SABR) for Adolescent and Young Adult Malignancies

**DOI:** 10.7759/cureus.66890

**Published:** 2024-08-14

**Authors:** Raj Singh, Sophia Bishop, Jan Jenkins, Joanne Davis, Rituraj Upadhyay, Christopher McLaughlin, Sanjeev Sharma, Sujith Baliga, Joshua D Palmer

**Affiliations:** 1 Department of Radiation Oncology, The Ohio State University Comprehensive Cancer Center, Columbus, USA; 2 Department of Radiation Oncology, The Radiosurgery Society, San Jose, USA; 3 Clinical Programs, The Radiosurgery Society, San Mateo, USA; 4 Department of Radiation Oncology, University of Virginia School of Medicine, Charlottesville, USA; 5 Department of Radiation Oncology, St. Mary's Medical Center, Huntington, USA

**Keywords:** adolescent and young adult, overall survival, toxicity, local control, local recurrence, metastases, stereotactic ablative radiation therapy

## Abstract

Background: There are limited studies examining local control (LC) and overall survival (OS) following stereotactic ablative radiation therapy (SABR) for adolescent and young adult (AYA) populations/histologies with local recurrences or metastatic disease.

Methods: The RSSearch® Patient Registry, an international SABR registry, was evaluated for AYA patients treated with SABR. AYA patients with adult histologies/primaries were excluded. Kaplan-Meier analyses were employed to characterize LC and OS following SABR. Potential prognostic factors were assessed with log-rank tests for initial univariate analysis (UVA). For multivariate analyses (MVA), a Cox proportional hazards multivariate model was utilized.

Results: A total of 19 AYA patients with 39 lesions treated with SABR were identified and included in the analysis. Four lesions (10.3%) were treated with SABR for primary tumor recurrence and 35 lesions were treated for metastatic disease. The median patient age was 34 years (range: 16-39 years). Common lesion locations included lung (11 lesions; 28.2%), non-spinal bone (nine lesions; 23.1%), and spine (six lesions; 15.4%). The median biological effective dose (BED_10_) was 61.5 Gy (range: 26.4-180). One-year LC and OS following SABR were 77.7% (95% CI: 58.5-88.7) and 72.7% (95% CI: 46.3-87.6), respectively. On UVA, BED_10_ ≥ 60 Gy was associated with superior one-year LC (94.4% vs. 47.6%; p<0.0001) as were sarcoma primaries (two-year LC: 92.3% vs. 42.2%;p = 0.0002). Central nervous system (CNS) primaries had significantly poorer one-year LC (20% vs 87.5%; p<0.0001) as well as spinal metastases (33.3% vs. 87.0%; p<0.0001). On MVA, BED_10_ < 60 Gy was associated with inferior LC (hazard ratio (HR) = 5.51;p = 0.01) with sarcoma primaries associated with superior LC (HR = 0.04;p = 0.008).

Conclusion: SABR with BED_10_ ≥ 60 Gy resulted in durable LC for AYA patients, particularly those with sarcoma primaries, though poor outcomes were noted in metastatic CNS malignancies.

## Introduction

In 2023, roughly 15,000 children and adolescents were diagnosed with cancer, and approximately 1,600 will die from their disease in the United States [1]. Recurrent and metastatic diseases in pediatric and adolescent and young adult (AYA) patients are especially challenging to treat and are associated with poor outcomes and significant morbidity [2,3]. Historically, the role of radiation therapy for metastatic and/or recurrent disease was limited to whole lung irradiation for particular histologies or palliative conventional fractionated re-irradiation. However, as treatment techniques have improved over time, more aggressive strategies are more routinely employed with local treatment of few or all metastatic sites, especially for patients with an initial response to systemic therapy, and have been associated with improved outcomes [4].

Stereotactic ablative radiation therapy (SABR) is an advanced radiation therapy technique that delivers high biologically effective doses (BED) of radiation to tumors with rapid dose fall-off, which may have the potential benefit of providing superior local control (LC) vs. conventional radiation therapy techniques, particularly for histologies traditionally considered be radioresistant such as chondrosarcomas and osteosarcomas [5,6]. In adult patients with oligometastatic disease, SABR has been shown to potentially improve overall survival (OS) in combination with standard-of-care systemic therapy with limited morbidity and excellent LC and also results in improved and durable palliation of pain [7-9]. In the setting of AYA populations, only a limited number of prospective and retrospective series have reported on clinical outcomes of SABR for the treatment of recurrent and metastatic AYA tumors [10-13]. In this study, we aimed to evaluate real-world outcomes with SABR for local recurrence or metastases from AYA malignancies/histologies and characterize LC, OS, and treatment-related toxicities using the RSSearch® Patient Registry.

## Materials and methods

The Radiosurgery Society® RSSearch® Patient Registry (NCT01885299) is a platform-agnostic SABR registry. Centers treating patients with SABR and/or SRS are able to join the registry. Each center is required to obtain local Institutional Review Board/Ethics Committee (IRB/EC) approval prior to the input of anonymized patient data. Further details regarding the registry can be found from prior analyses utilizing the RSSearch® Patient Registry [14-16].

The registry was queried for AYA patients, ages <39 years, with either extracranial metastases or locally recurrent extracranial disease treated with SABR. All patients included in this analysis were treated on the CyberKnife® platform. Patients eligible for inclusion were those of 39 years of age or less with non-breast and prostate adenocarcinoma histologies (22 patients with breast cancer and 25 patients with prostate cancer) with either extracranial metastases or locally recurrent disease, with available clinical and radiographic follow-up data for analysis of LC and overall survival (OS). Other information examined collected was dose/fractionation schedule, Karnofsky Performance Score (KPS), and location(s) of metastases or local recurrences. All others not meeting the above inclusion criteria were also excluded from the analysis.

Follow-up and endpoints

Patients were followed per participating standard operating practices and commonly comprised clinical follow-ups with relevant imaging every three months, with the specific imaging depending on the treated site. LC was defined as the number of months from the completion of SABR to the last radiographic follow-up with stable disease, partial response, or complete response achieved. We defined OS as the number of months from the completion of SABR to the date of the last follow-up or death. At each clinical follow-up, any treatment-related toxicities were assessed for and, subsequently, logged with grading per Common Terminology Criteria for Adverse Events (CTCAE) version 3.0 or 4.0.

Statistical analysis

STATA version 14.0 (StataCorp, College Station, TX) was employed for statistical analyses. We performed univariate analyses first with Kaplan-Meier analysis and log-rank tests. A forward entry parsimonious method with criteria of independent variables that approached statistical significance (p ≤ 0.10) was employed to determine which variables were included in multivariate (MVA) Cox proportional hazard models. For comparison between difference dose/fractionation schedules, biologic effective doses (BED_10_) were calculated with an assumed alpha-beta ratio of 10. We defined patients treated with "dose escalated" plans as those that received a BED_10_ ≥ 60 Gy (based on the median/dose fractionation in our study of 35 Gy/5 fractions). Logistic regression was employed to examine for any relationship between dose escalation and the incidence of toxicities attributable to SABR. Statistical significance was defined as a p-value ≤ 0.05.

## Results

Patients that were identified for analysis can be found in Table 1, with information on both lesion and treatment characteristics. There were 19 patients identified with 39 lesions treated with SABR. The most common histologies of treated lesions were sarcomas, including chondrosarcoma (nine lesions), alveolar soft part sarcoma (four lesions), osteosarcoma (three lesions), and Ewing sarcoma (two lesions) and central nervous system (CNS) malignancies spread to the spine (five lesions; 12.8%, including anaplastic ependymoma and astrocytoma). Traditionally presumed radioresistant histologies, including chondrosarcoma, alveolar soft part sarcoma, osteosarcoma, synovial sarcoma, and sarcoma, NOS were included in subgroup analyses for purposes of the analysis of LC (18 lesions). Four lesions (10.3%) were treated for primary tumor recurrence, with the remainder treated for metastatic disease. The median patient age was 34 years (range: 16-39), and the median KPS was 90% (range: 60-100). The most common locations of lesions that were treated with SABR were the lung (11 lesions; 28.2%), non-spinal bone (nine lesions; 23.1%), and spine (six lesions; 15.4%). The median gross tumor volume (GTV) was 12.4 cc (range: 0.7-233.5). With regards to planning characteristics, the median prescription dose was 35 Gy (range: 12-60) delivered in a median of five fractions (range: 1-5) with a median BED_10 _of 61.5 Gy (range: 26.4-180).

**Table 1:** Patient, lesion, and radiotherapy planning characteristics. GTV: gross tumor volume; BED: biological effective dose; KPS: Karnofsky performance score; NOS: not otherwise specified.

Variable	
Patients (lesions) treated	19 patients (39 lesions)
Gender	
	Male: 12 patients
	Female: 6 patients
	Unknown: 1 patient
Median age (years) (range)	34 years (16-39)
Race	
	Caucasian: 15 patients
	African-American: 1 patient
	Latino/Hispanic: 1 patient
	Other/unknown: 2 patients
Median initial KPS (range)	90% (60%-100%)
Median GTV (cc) (range)	12.4 (0.7-233.5)
Primary histology	
	Chondrosarcoma, NOS: 9 lesions
	Alveolar soft part sarcoma: 4 lesions
	Anaplastic ependymoma: 4 lesions
	Osteosarcoma, NOS: 3 lesions
	Desmoplastic small round cell tumor: 3 lesions
	Ewing sarcoma: 2 lesions
	Malignant melanoma, NOS: 2 lesions
	Synovial sarcoma, NOS: 1 lesion
	Leiomyosarcoma, NOS: 2 lesions
	Renal cell carcinoma, sarcomatoid: 1 lesion
	Anaplastic astrocytoma: 1 lesion
	Sarcoma, NOS: 1 lesion
	Other/not specified malignancy: 6 lesions
Metastatic sites treated	
	Lung: 11 lesions
	Non-spinal bone: 9 lesions
	Spine: 6 lesions
	Retroperitoneum/peritoneum: 2 lesions
	Base of tongue: 2 lesions
	Lymph nodes: 2 lesions
	Liver: 1 lesion
	Accessory sinus: 1 lesion
	Other/unknown: 5 lesions
Median number of fractions (range)	5 (1–5)
Median prescription dose (Gy) (range)	35 (12-60)
Most common dose/fractionation schedule	35 Gy/5 fractions
Median BED_10_ (Gy_10_) (range)	61.5 (26.4-180)

**Figure 1:**
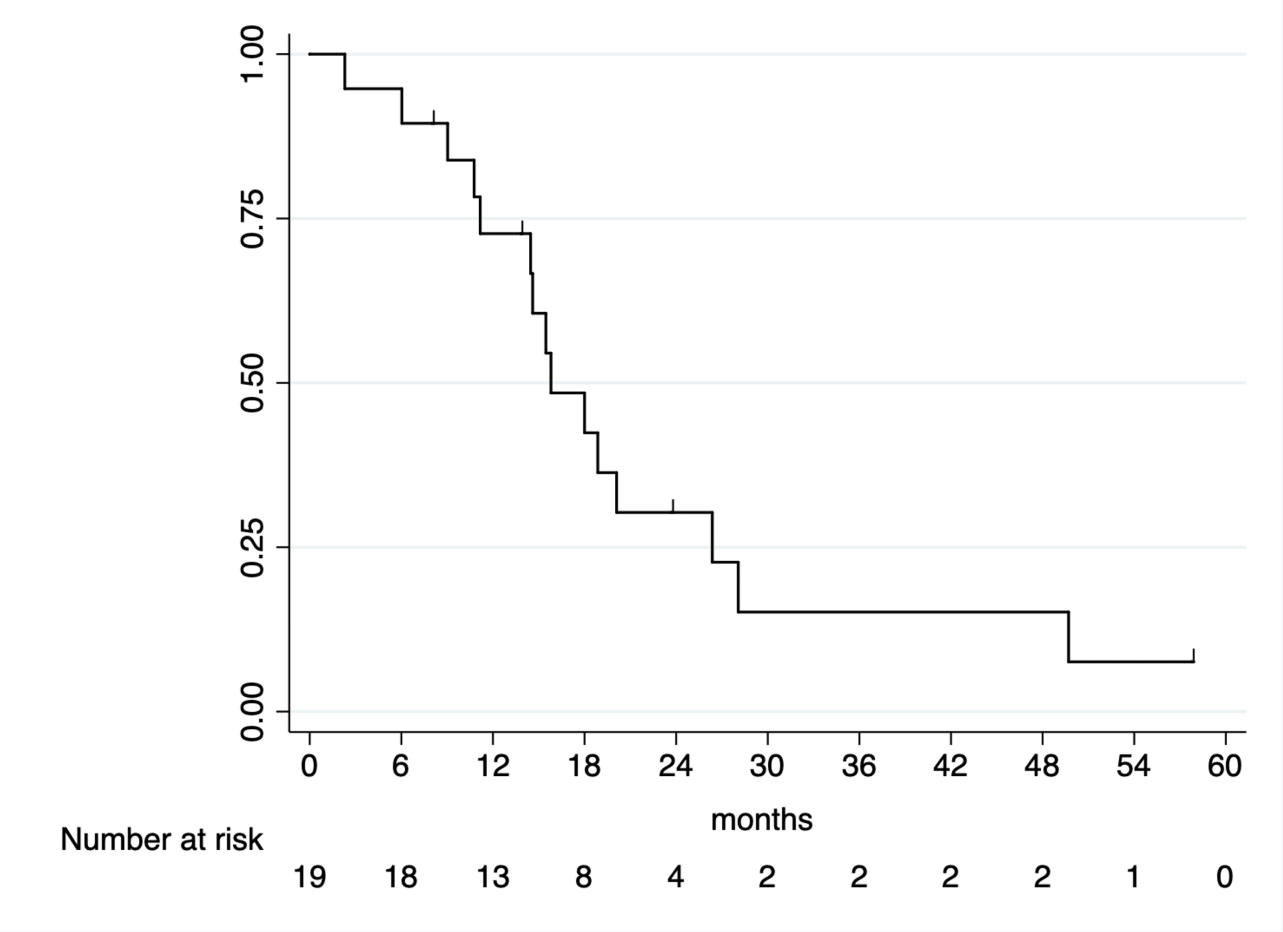
Kaplan-Meier curve of OS following SABR. SABR: stereotactic ablative radiation therapy, OS: overall survival.

One- and two-year OS rates following SABR were 72.7% (95% CI: 46.3-87.6) and 30.3% (95% CI: 11.2-52.2%), respectively (Figure 1). On examination of characteristics including primary tumor histology, lesion location, KPS, and age, no prognostic factors showed a correlation with OS on UVA; therefore, MVA was not performed (Table 2).

**Table 2:** One-year OS results stratified by prognostic factors on UVA. OS: overall survival; CI: confidence interval; CNS: central nervous system; UVA: univariate analysis; KPS: Karnofsky Performance Score.

Variable	One-year OS (95% CI)	p-value
Age		0.94
34 years or older	70% (32.9-89.2%)	
Less than 34 years	77.8% (36.5-93.9%)	
Lung vs. non-lung lesions		0.97
Lung lesions	78.6% (47.3-92.5%)	
Non-lung lesions	53.3% (6.8-86.3%)	
Initial KPS		0.85
90%-100%	71.2 % (41.4-88.4%)	
<90%	75% (12.8-96.1%)	
Histology		0.85
Sarcoma	60.0% (12.6-88.2%)	
Non-sarcoma	78.6% (47.3-92.5%)	
Primary site		0.81
CNS primary	100% (N/A)	
Non-CNS primary	69.3% (41.2-86.0%)	

**Figure 2:**
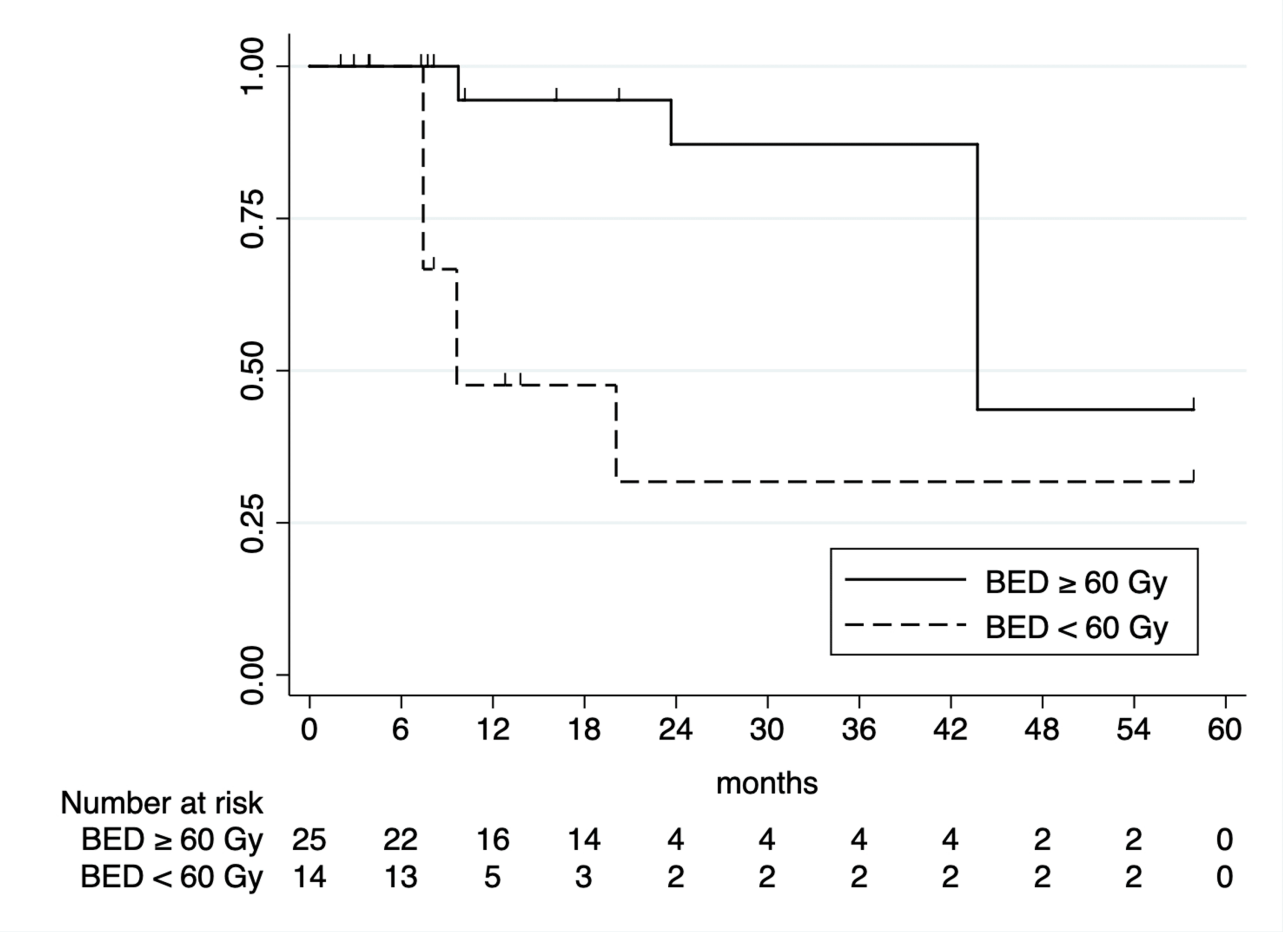
Kaplan-Meier curve of local control by BED10.

**Figure 3:**
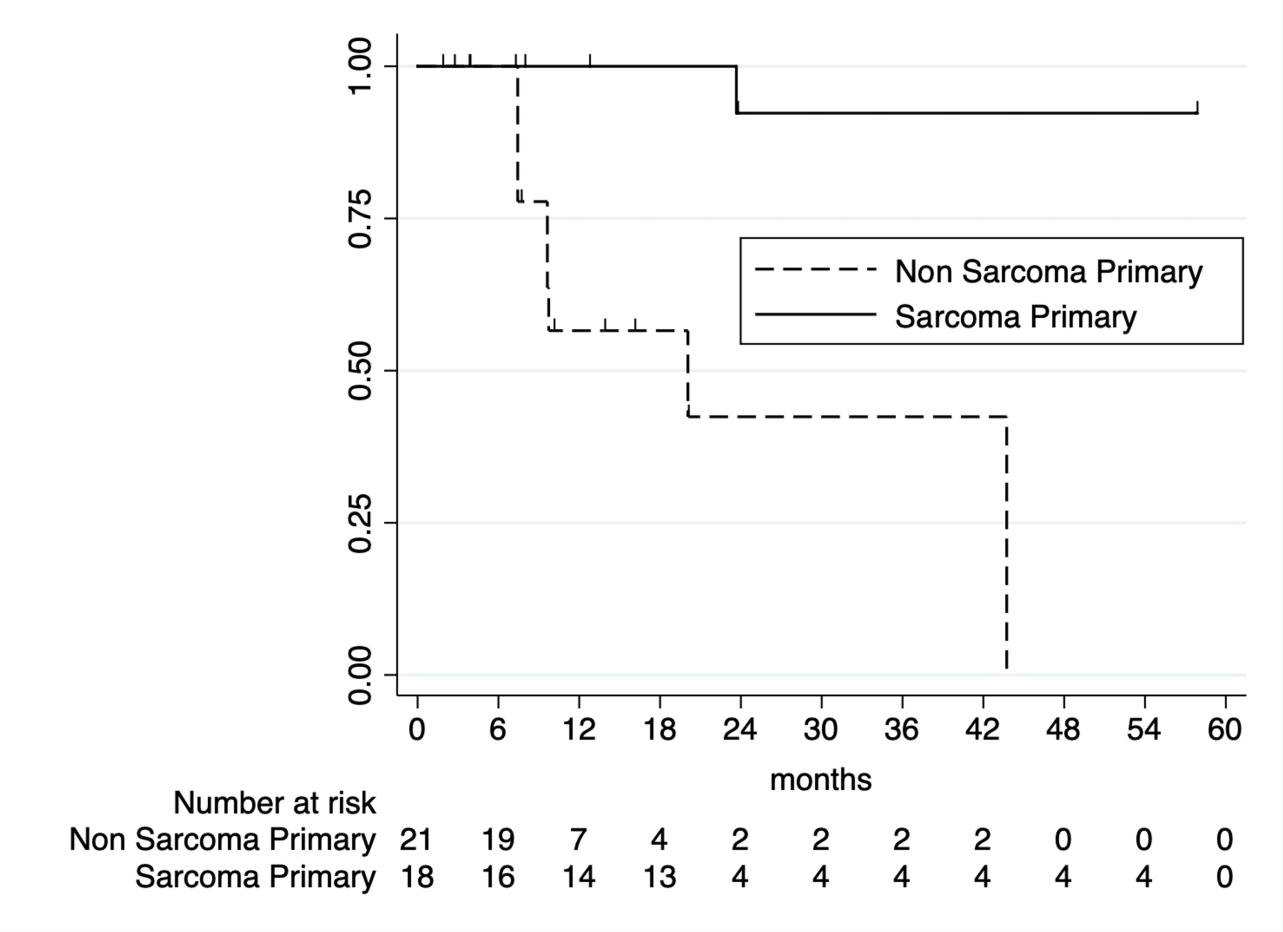
Kaplan-Meier curve of local control by sarcoma vs. non-sarcoma primary histology.

**Figure 4:**
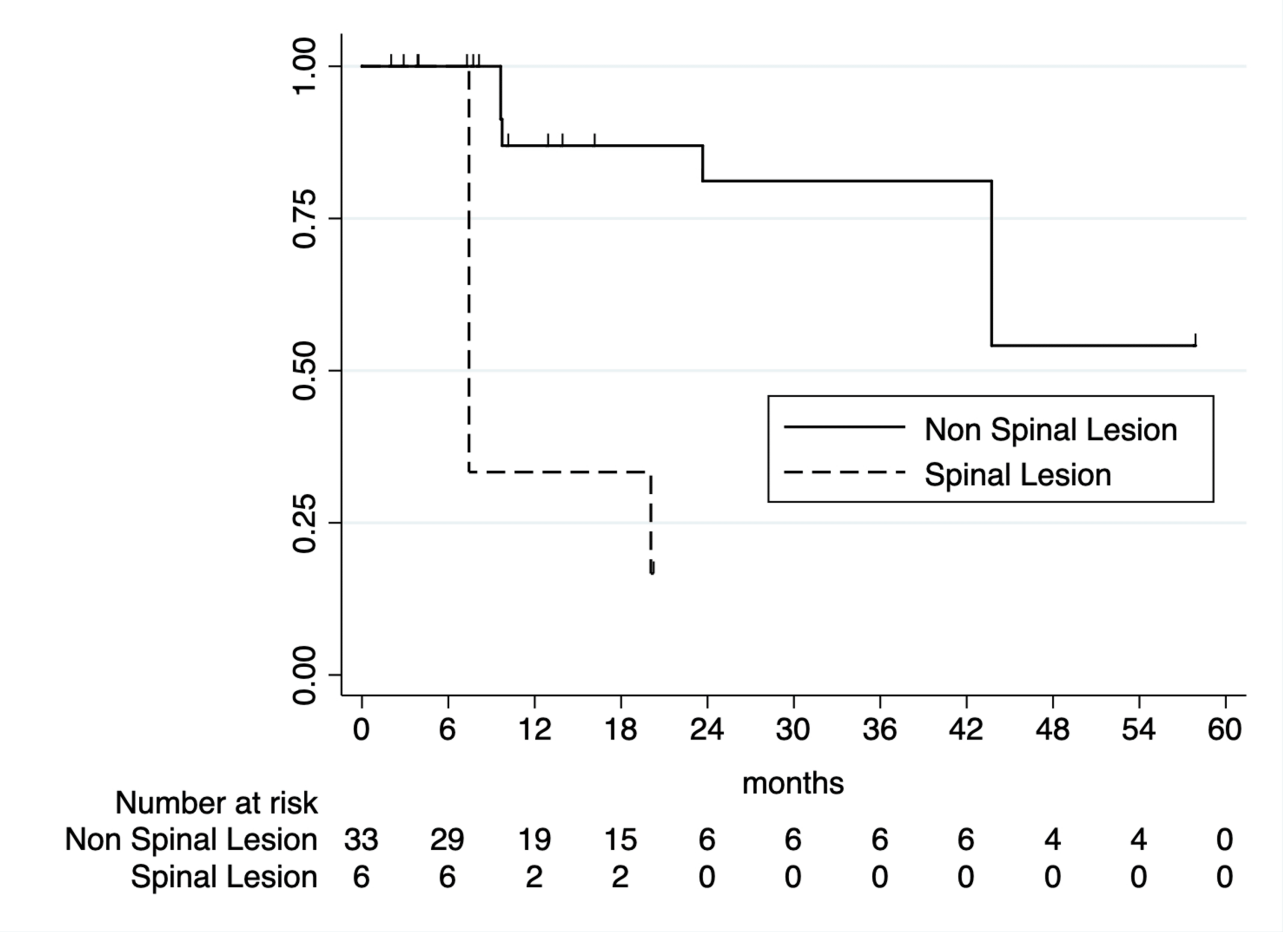
Kaplan-Meier curve of local control by spinal vs. non-spinal lesion.

One- and two-year LC rates following SABR were 77.7% (95% CI: 58.5-88.7%) and 68.2% (95% CI: 46.9-82.4%), respectively. Initial UVA analysis identified BED_10_ ≥ 60 Gy as being significantly associated with improved LC (one-year LC of 94.4% vs. 47.6%; p < 0.0001; Figure 2). Primary tumor histology was associated with two-year LC, with sarcoma primaries associated with superior two-year LC (92.3% vs. 42.2%; p = 0.0002; Figure 3). CNS primaries were associated with significantly poorer one-year LC (87.5% vs. 20%; p < 0.0001). Additionally, spinal lesions were associated with significantly inferior one-year LC following SABR (33.3% vs. 87.0%; p < 0.0001; Figure 4). Of note, 5/6 spinal metastases were from CNS primaries, with all having local failures; as such, due to collinearity, CNS primaries were unable to be included on MVA. On MVA, including BED_10_, spinal metastases, and sarcoma histologies, BED_10_ < 60 Gy was associated with inferior LC (hazard ratio (HR) = 5.51 (95% CI: 1.42-21.33); p = 0.01) with sarcoma primaries associated with superior LC (HR = 0.04 (95% CI: 0.004-0.45); p = 0.008).

With respect to treatment-related toxicities, nine of 39 treated lesions (8.5%) had side effects attributable to SABR. All were CTCAE Grade 1-2 with no relationship noted between the incidence of toxicities and dose escalation (p = 0.27).

## Discussion

For adult patients with oligometastatic cancer, SABR has been shown to improve disease control in combination with standard-of-care systemic therapy and also with improved palliative outcomes as well. Similarly, SABR has significant potential benefits for AYA patients with cancer, including providing durable LC with limited morbidity, improved palliation of symptoms, and shorter treatment sessions that may minimize interruptions in systemic therapy as compared to conventionally fractionated radiation therapy. Our study noted acceptable LC with minimal treatment-related toxicities and notably found a LC benefit with dose escalation with BED_10_ exceeding 60 Gy without a subsequent increase in toxicity. Notably, sarcoma histologies that have historically been considered radioresistant were in fact found to have the most favorable LC outcomes following SABR, with metastases from CNS primaries having the poorest LC.

Our findings compare favorably to those previously reported in the literature on SABR for pediatric and AYA patients with locally recurrent or metastatic cancer. There have been wide ranges in one-year LC that have been reported following SABR ranging anywhere from 54 to 94% [10-13,17-20]. The largest systemic review and meta-analysis to date on this topic included 142 patients with 217 lesions across nine publications examining SABR for pediatric and AYA patients. The most common histologies included osteosarcoma, Ewing sarcoma, soft-tissue sarcoma, and neuroblastoma. Following SABR, the estimated one-year and two-year LC rates were 83% and 74%, respectively. Of note, a dose-response was noted with superior LC for studies utilizing higher BED_10_ for sarcoma-predominant studies, similar to the findings of our study that noted excellent LC among sarcoma lesions (two-year LC of 92.3% vs. 42.3%) [20]. A recently published institutional series by Upadhyay et al. similarly noted superior one-year LC among sarcoma vs. non-sarcoma histologies (95.7% vs 86.5%, p = 0.01) [12].

Similarly, other studies have also noted poorer LC for patients with CNS primaries and/or spinal metastases/lesions. Tinkle et al. reported on outcomes following SABR for 55 children and AYA patients with 107 non-CNS lesions treated with SABR. Notably, of the 27 lesions that were noted to have local progression, seven were paraspinal or vertebral body lesions [13]. For spinal lesions, organs-at-risk (i.e., spinal cord and the cauda equina) led to poorer target-volume coverage, particularly for those with paraspinal disease and with epidural extension. This has also been noted to be a challenge with respect to adult patients with metastatic disease, as treating gross disease adjacent to the spinal cord can limit ablative dosing [21]. One limitation of our study is the lack of imaging and detailed assessment of the SABR treatment plans to evaluate target coverage and assessment of local progression for individual cases. Dosimetric analyses and imaging will be important for future studies involving AYA cancer patients, given the concerns about late toxicities and the careful consideration that must be taken when evaluating patients, both AYA and adults, for spine SABR. 

Another aim of our analysis was to evaluate the optimal dose and fractionation schedule for SABR. We noted superior one-year LC with SABR plans utilizing BED_10_ exceeding 60 Gy (94.4% vs. 47.6%). One of the first series on SABR for pediatric metastatic sarcomas noted two local failures among 14 cases, with both cases having received a BED_10_ < 60 Gy [18]. A more recent larger series of 88 lesions treated with SABR found 9/10 local failures occurred in patients that received less than 40 Gy/five fractions (corresponding to a BED_10_ of 72 Gy) [19]. Upadhyay et al. also noted improved one-year LC outcomes for patients receiving a BED_10_ > 48 Gy (100% vs 91.2%, p = 0.001) [12]. Similarly, meta-analyses have also noted a dose-response among sarcoma predominant studies with every 10 Gy increase in BED_10_, resulting in an approximately 5% improvement in two-year LC [20]. Clinical trials evaluating higher BED_10_ doses in children and AYA patient cohorts have not generally been studied due to concerns for long-term toxicity risk and should only be considered within the oversight of a clinical trial. As the delivery of SABR expands and more uniform guidelines develop for AYA patients with cancer, assessment of dose and correlation with tumor control and toxicities will be critical to optimize this treatment approach.

Limitations

Limitations to these data are that RSSearch includes its heterogeneity in patient and tumor characteristics, SABR treatment planning and delivery techniques, and various follow-up schedules defined at the local treatment center. The database provided limited information on concurrent treatments, such as chemotherapy, immunotherapy or surgery, and lack imaging and detailed dosimetric analysis of SABR plans. However, these real-world data can be used to evaluate predictive factors in a multi-institutional setting, which can then be considered for future prospective clinical trials.

## Conclusions

In this study, SABR was well-tolerated and resulted in durable LC. When feasible, dose escalation with BED_10_ ≥ 60 Gy improved LC without significant toxicity. CNS primaries and spinal metastases had poor outcomes, highlighting the need for alternate/synergistic treatment strategies. Future studies examining the impact of histology for tailored dose/fractionation selection should be considered.
